# Replacement of the bladder: Any news on the horizon?

**DOI:** 10.14440/bladder.0226

**Published:** 2025-08-22

**Authors:** Richard E. Hautmann

**Affiliations:** Department of Urology, University of Ulm, Neu-Ulm 89231, Germany

Patients requiring replacement of their bladder currently have only one option for urinary reconstruction using a vascularized segment of intestine, mostly the ileum, which can expose them to multiple complications. These include infections, stones, renal dysfunction, metabolic issues, etc. The risk of short- and long-term complications, including reoperation, is substantial and will remain for the rest of the patient. Consequently, a non-intestinal derived diversion, i.e., avoidance of the intestinal anastomosis, would be a major progress. In 2025, several options have been made available and deserve mention:

## 1. Bladder transplantation

Recently, Gargollo *et al*.^1^ have demonstrated that urinary bladder vascularized composite allograft transplantation was technically and anatomically feasible in two adult cadavers. At present, a phase 1 clinical trial studying the safety and feasibility of concomitant renal and urinary bladder vascularized composite allograft transplantation is in progress at the Mayo Clinic, Rochester, USA. However, several high hurdles remain due to the need for immunosuppression and to the atonic nature of the construct, particularly in patients who likely still require intermittent catheterization.

In 2023, Nassiri *et al*. at USC published pre-clinical studies on preparation for the first in-human bladder transplant.^2^

Successful robotic vascularized composite bladder allograft auto-transplantation was achieved in two porcines, one cadaver, and three brain-dead research donors. In the heart-beating research donors, console time decreased with successive surgeries, and visual inspection revealed healthy revascularized autografts with prompt, global indocyanine green immunofluorescence uptake. In one heart-beating donor who was hemodynamically maintained for 12 h postoperatively, reinspection confirmed excellently maintained global vascularized composite bladder allograft vascularity and bladder mucosal integrity.

This experience represents the essential pre-clinical work required to move toward the first-in-human trial of bladder transplantation, performed under a UNOS-approved genitourinary vascularized composite bladder allograft program (NCT No. 05462561).^2^

In the foreseeable future, the indication for bladder transplantation will be limited, since patients require lifelong immunosuppression and have the associated adverse effects. In addition, the lack of neural connections allows only for the replacement of the storage function of the bladder, whereas voiding will still need catheterization.

## 2. Artificial urinary diversion systems

The “off-the-shelf” possibility to replace the bladder is intriguing. Over the last seven decades, multiple attempts have been made to develop an artificial bladder. Since the 1960s, surgeons, scientists, and the industry worldwide have been working on new systems. However, despite progress in technology and knowledge, the outcome continues to be discouraging (refer to several review articles in this regard for more information^3^,^4^).

## 3. Regenerative medicine in bladder reconstructive surgery (tissue engineering)

In the short term, a wide array of materials proved to be suitable and could withstand the corrosive effect of urine. However, encrustation occurred due to deposition of minerals, fibrous capsules around the implanted material, infection, and hydronephrosis, and even renal failure invariably occurred. These failures were primarily due to incomplete material mechanical resilience manifested by residual urine or urinary leaks along suture lines. The combination of limited prompt angiogenesis—common to all tissue-engineered constructs—and the cytotoxic effects of urine specific to the urinary system accelerates fibrous capsule formation and contributes to the failure of tissue-engineered bladders. To date, despite great research endeavors made in tissue engineering, urologists do not yet have a reliable off-the-shelf construct to offer patients who need bladder substitution.^5^

Sloff *et al*.^6^ examined the literature to elucidate why we have not yet reached the ultimate goal of a tissue-engineered bladder. Although their search strategy was comprehensive, it was limited to English literature, and they used bladder volume measurement as the primary predictive variable for successful outcome. Nevertheless, this analysis is unique not because it lists the variability in the conducted research, but rather because it encapsulates the evolution of the bladder tissue engineering field and may set the course for a new era in this research area.^6^

## 4. Status of the ileal neobladder (INB)

Advantages and disadvantages of the INB are well understood. However, based on the current progress, INB stands as the best option for bladder replacement, at least for the foreseeable future. Nevertheless, multiple concerns regarding the INB remained unaddressed.

The term “INB” is the abbreviation of “Ileal Neobladder,” which gradually becomes synonymous with “orthotopic reconstruction” or “bladder replacement.”^7^ While the INB approach has gained broader popularity over other equivalent treatment methods, its adoption is met with a declining trend. Groeben *et al*.^8^ have analyzed the nationwide German hospital database and the Nationwide Inpatient Sample in the US from 2006 to 2014. The share of continent diversion in the US remained stable, being as low as 7%, while the share decreased from 36.8% to 29.2% in Germany. In a Germany-based nationwide trend analysis from 2005 to 2021, Klemm *et al*.^9^ have also shown that continent diversion is losing its momentum. However, it must be acknowledged that these numbers come despite exceptionally high numbers at large, tertiary referral centers in Germany and the US. Potential reasons for the decline of INB adoption are the increase in elderly patients, surgical volume of the center, imperfect functional outcomes of the INB, or the technical challenges with a steep learning curve for the robotic adoption of (intracorporeal) INB.^10^ Furthermore, surgeon preference and economic considerations could contribute to this decline.

Since Tizzoni and Foggi^11^ conceived the idea of bladder replacement in 1888, before the concepts of urology or urologists appeared, numerous efforts have been made to accomplish this goal.^7^ To construct a bladder as good as or even better than the natural organ, the question still lingers: When will we get there? The most likely answer in 2025 is probably never.

## Conflict of interest

Richard E. Hautmann is an Honorary Editor-in-Chief of this journal. The author declared that he has no known competing financial interests or personal relationships that could have influenced the work reported in this paper.

## References

[1] Gargollo PC Ahmed ME Prieto M Feasibility study of vascularized composite urinary bladder allograft transplantation in a cadaver model. J Urol 2021;206 115–123. doi: 10.1097/JU.0000000000001699 33683936 10.1097/JU.0000000000001699

[2] Nassiri N Cacciamani G Gill IS Robotic bladder autotransplantation:Preclinical studies in preparation for first-in-human bladder transplant. J Urol 2023;210 600–610. doi: 10.1097/JU.0000000000003620 37681535 10.1097/JU.0000000000003620

[3] Chua ME Farhat WA Ming JM McCammon KA Review of clinical experience on biomaterials and tissue engineering of urinary bladder. World J Urol 2020;38 2081–2093. doi: 10.1007/s00345-019-02833-4 31222507 10.1007/s00345-019-02833-4

[4] Cosentino M Gaya JM Palou J Villavicencio H Alloplastic bladder substitution:Are we making progress?. Int Urol Nephrol 2012;44 1295–1303. doi: 10.1007/s11255-012-0249-2 22821051 10.1007/s11255-012-0249-2

[5] Farhat WA Tissue engineering of the bladder –when will we get there?. J Urol 2014;192 1021–1022. doi: 10.1016/j.juro.2014.07.079 25046604 10.1016/j.juro.2014.07.079

[6] Sloff M Simaioforidis V de Vries R Oosterwijk E Feitz W Tissue engineering of the bladder-reality or myth?A systematic review. J Urol 2014;192 1035–1042. doi: 10.1016/j.juro.2014.03.116 24769032 10.1016/j.juro.2014.03.116

[7] Hautmann RE Moll F Etymology of the neobladder. Urologie 2025;3 269–275. doi: 10.1007/s00120-025-02529-1 10.1007/s00120-025-02529-1 39998624

[8] Groeben C Koch R Baunacke M Urinary diversion after radical cystectomy for bladder cancer:Comparing trends in the US and Germany from 2006 to 2014. Ann Surg Oncol 2018;25 3502–3509. doi: 10.1245/s10434-018-6381-1 29468604 10.1245/s10434-018-6381-1

[9] Klemm J Fisch M Laukhtina E Dahlem R Shariat SF Vetterlein MW Continent diversion is losing its momentum:A nationwide trend analysis from Germany 2005-2021. BJU Int 2023;133 154–157. doi: 10.1111/bju.16215 37899645 10.1111/bju.16215

[10] Duwe G Kamal MM Wiesmann C Temporal trends in urinary diversion among patients undergoing radical cystectomy between 1986 and 2022:Experience at the Univerity Medical Center Mainz with 2224 Cases. Ann Surg Oncol 2024;31 7220–7228. doi: 10.1245/s10434-024-15730-x 38969859 10.1245/s10434-024-15730-x PMC11413057

[11] Tizzoni G Foggi A Die Wiederherstellung der Harnblase. Experimentelle Untersuchungen. Centralblatt für Chirurgie 1888;15 921–924

